# PREDICTORS OF PRESSURE ULCER DEVELOPMENT AMONG SENIORS DURING EMERGENCY TRANSFERS TO HOSPITAL EMERGENCY DEPARTMENT

**DOI:** 10.1093/geroni/igz038.1683

**Published:** 2019-11-08

**Authors:** Colin Reid, Hannah Jiwani, Kaitlyn C Tate, Greta G Cummings

**Affiliations:** 1 University of British Columbia, Kelowna, British Columbia, Canada; 2 University of Alberta, Edmonton, Alberta, Canada

## Abstract

Emergency transfers of seniors in long-term care facilities (LTCF) aged >65 to hospital emergency departments (ED) are common and carry with them risks that can lead to less-than-optimal quality of care and quality of life. Pressure ulcers are one such risk. We used data from the Older Persons Transitions in Care (OPTIC; N=637) study, conducted in two Canadian provinces in 2011 and 2012, to assess potential predictors of pressure ulcer development between the time that a resident is transported to the ED until the time they return to their original nursing home. Step-wise binary logistic regression was employed to identify predictors of pressure ulcer development during the transition. Potential predictors included length of transition, inpatient status, demographic, health variables (including incontinence). Among the 335 residents for whom we were able to gather new pressure ulcer data, 56 (16.7%) were identified as having developed new skin wounds upon return to the LTCF. Transitions from ED admission to return to LTCF averaged 106.7 hours (sd=143.6) with a median of 50.0 hours. Length of transition and whether the resident spent time as an inpatient emerged as the only predictors: longer transition times and spending time as an inpatient predict development of bed sores. These results speak to the need for improved monitoring and treatment of skin wounds during emergency transitions of older adults from LTCF.

